# Blood Lead Level and Risk of Asthma

**DOI:** 10.1289/ehp.7453

**Published:** 2005-03-03

**Authors:** Christine L.M. Joseph, Suzanne Havstad, Dennis R. Ownby, Edward L. Peterson, Mary Maliarik, Michael J. McCabe, Charles Barone, Christine Cole Johnson

**Affiliations:** ^1^Department of Biostatistics and Research Epidemiology, Henry Ford Health System, Detroit, Michigan, USA; ^2^Allergy–Immunology Section, Medical College of Georgia, Augusta, Georgia, USA; ^3^Department of Environmental Medicine, University of Rochester School of Medicine, Rochester, New York, USA

**Keywords:** asthma, atopy, environment, immunoglobulin E, incidence, lead poisoning, racial disparity

## Abstract

Asthma and lead poisoning are prevalent among urban children in the United States. Lead exposure may be associated with excessive production of immunoglobulin E, possibly increasing asthma risk and contributing to racial disparities. The objective of this study was to examine racial differences in the association of blood lead level (BLL) to risk of developing asthma. We established and followed a cohort prospectively to determine asthma onset, using patient encounters and drug claims obtained from hospital databases. Participants were managed care enrollees with BLL measured and documented at 1–3 years of age. We used multiple variable analysis techniques to determine the relationship of BLL to period prevalent and incident asthma. Of the 4,634 children screened for lead from 1995 through 1998, 69.5% were African American, 50.5% were male, and mean age was 1.2 years. Among African Americans, BLL ≥5 and BLL ≥10 μg/dL were not associated with asthma. The association of BLL ≥5 μg/dL with asthma among Caucasians was slightly elevated, but not significant [adjusted hazard ratio (adjHR) = 1.4; 95% confidence interval (CI), 0.7–2.9; *p* = 0.40]. Despite the small number of Caucasians with high BLL, the adjHR increased to 2.7 (95% CI, 0.9–8.1; *p* = 0.09) when more stringent criteria for asthma were used. When compared with Caucasians with BLL < 5 μg/dL, African Americans were at a significantly increased risk of asthma regardless of BLL (adjHR = 1.4–3.0). We conclude that an effect of BLL on risk of asthma for African Americans was not observed. These results demonstrate the need for further exploration of the complex interrelationships between race, asthma phenotype, genetic susceptibilities, and socioenvironmental exposures, including lead.

Strategies for the prevention of asthma remain elusive [Centers for Disease Control and Prevention (CDC) 2002; Hartert and Peebles 2000]. In the United States, asthma morbidity is highest among minorities and persons of low socioeconomic status (SES) (Grant et al. 2000; Miller 2000). African-American and Hispanic children in the United States have emergency department (ED) and hospitalization rates for asthma that are two to four times higher than that observed in Caucasian children, and African-American asthma mortality rates can be four times higher (CDC 2002; Grant et al. 2000).

Lead poisoning is a serious environmental health hazard for U.S. children of minority status and low SES (CDC 2001). The effects of lead poisoning include delayed cognitive development, permanent learning disabilities, and behavior problems (Lanphear et al. 1998, 2002; Needleman 1998). According to national surveys, African-American children were found to have blood lead levels (BLL) four times higher than those of Caucasians after controlling for income and urban status, and were seven times more likely than Caucasians to require medical evaluation for lead poisoning (CDC 2001). Although federal guidelines recommend intervention at BLL ≥10 μg/dL, adverse outcomes have been demonstrated at lower levels (Bernard and McGeehin 2003).

The epidemiology of pediatric asthma and that of lead poisoning are strikingly similar (Hartert and Peebles 2000; Lanphear et al. 1998). Both are prevalent among minority children, and elements in the physical environment increase risk of disease (Hartert and Peebles 2000; Lanphear et al. 1998; Rosenstreich et al. 1997). Low SES and residing in an urban setting are associated with increased risk for both conditions (Bernard and McGeehin 2003; Miller 2000).

Published analyses suggest that lead exposure may result in alterations to immune system components known to be associated with asthma (Lutz et al. 1999; Sun et al. 2003). Lead has been associated with the increased production of total immunoglobulin E (IgE), which is also observed in atopic and nonatopic individuals with asthma (Beeh et al. 2000; Romanet-Manent et al. 2002).

The immunotoxic or immunomodulatory effects of lead have been demonstrated recently in animal models, and include impaired host resistance to infections and an enhancement of alloantigenic-specific T-cell proliferation by altering antigen processing and presentation (Gupta et al. 2002; McCabe et al. 2001). Both Lutz et al. (1999) and Sun et al. (2003) reported an association between lead and increased IgE in studies of young children.

We hypothesize that differential risk of lead poisoning among urban minority children may contribute to increased risk of asthma in this population. The overall goal of this analysis was to use encounter and claims data to examine relationships between BLL and development of asthma, by race.

## Materials and Methods

### Design.

The methods of this study were approved by the Henry Ford Health System (HFHS) institutional review board. The base study population was enrollees of a large, nonprofit managed care organization (MCO) in southeastern Michigan served by physicians in a staff model medical group. The MCO has a stable population of > 250,000 with a mean enrollment of 9.3 years. The MCO enrollment database and the associated laboratory database (all lead screens are sent to a single central laboratory) were linked to identify children born on or between 1 January 1994 and 31 December 1997, enrolled in the MCO at time of birth, and with laboratory results for a lead screen performed between 1 January 1995 and 31 December 1998 (baseline BLL) [National Institute for Occupational Health and Safety (NIOSH) 1994]. When results existed for more than one lead screen for an individual child, the highest BLL recorded within the study ascertainment period was used as the baseline BLL. Usually this was the first BLL documented. The resulting data set was linked to the patient encounter database to obtain all ambulatory and inpatient visits, as well as demographic information, including child’s date of birth, race, and residential address. The pharmacy claims database provided information on drug claims for asthma medications [Health Employers Data Information System (HEDIS) 1999].

MCO enrollment and disenrollment dates were used to calculate the person-years that each child contributed to the cohort. Geographic information system (GIS; Mapping Solutions, LLC, Lansing, MI), a computer mapping and analysis technology capable of linking geographic with demographic information, was used in conjunction with patient address and census data (U.S. Census Bureau 2000). Each study participant was assigned the average income per person in the block group of residence (a subdivision of a census tract representing a city block) (Croner et al. 1996).

The method for obtaining birth weight for children in this cohort was approved by the State of Michigan Division for Vital Records and Health Statistics (Lansing, MI) in addition to the HFHS institutional review board. Identifiers for members of the study cohort were matched, at the State of Michigan Division for Vital Records and Health Statistics (Lansing, MI), to live birth records. Birth record identifier fields were not supplied to the researchers. Matches outside of the state were censored. The resulting match rate was 97.8%.

Blood samples were obtained by venipuncture, collected in EDTA tubes, and shipped at room temperature to the HFHS chemistry laboratory. Lead was measured in the blood using graphite furnace atomic absorption spectrophotometry, with detection limits of 1 μg/dL.

### Asthma definition.

We determined asthma status using enrollee encounter and pharmacy claims databases from HFHS. Two definitions of asthma were used. For definition 1, a child was considered to have asthma if the child had four or more asthma-medication–dispensing events in 12 months or met one or more of the following criteria within a 12-month period: one or more ED visits for asthma, one or more hospitalizations for asthma, or four or more outpatient visits for asthma with at least two asthma-medication–dispensing events. For definition 2, a child was considered to have asthma if the child had four or more asthma-medication–dispensing events in 12 months, and had one or more ED visits for asthma, one or more hospitalizations for asthma, or four or more outpatient visits for asthma with at least two asthma-medication–dispensing events (HEDIS 1999). These definitions are used by HEDIS to define an MCO population of patients with persistent asthma.

### Statistical analysis.

We determined period prevalence of asthma at baseline by taking the number of definition 1 or 2 asthma cases occurring from birth to 12 months after the index BLL and dividing it by the total number of children in the cohort at that time. Children who did not meet the criteria for asthma during this period were considered “asthma free” and used in the incident asthma analysis. Children meeting criteria for asthma during the postbase-line follow-up period were considered incident asthma cases. We calculated incidence density (ID) by taking the number of asthma cases that developed during the postbaseline follow-up period and dividing by the total number of person-years contributed to the cohort during this period (Pearce et al. 1998). Data were censored when a patient disenrolled from the MCO. Chi-square tests were used to compare sex, race, and baseline income proportions for children included in the study with those of children excluded from the study because of lack of a recorded BLL. We used a Wilcoxon rank-sum test to compare the distributions of BLL by race.

We assessed racial differences in the number of asthma cases using logistic regression for period prevalent cases and Cox proportional hazards for incident cases. The cutoffs used for BLL in these analyses were ≥5 μg/dL and ≥10 μg/dL. The association of period prevalent asthma to BLL was evaluated by computing odds ratios (ORs) and corresponding 95% confidence intervals (CIs). We evaluated the association of BLL to asthma incident cases by computing the hazard ratio (HR) along with the corresponding 95% CIs. We used a Cox proportional hazard to determine the independent association of BLL to asthma incident cases (Diggle et al. 1994). These models adjusted for average annual income per person, birth weight, and sex. Separate models were run for ≥5 μg/dL and ≥10 μg/dL for each race using < 5 μg/dL as the comparison group. In addition, separate models were run for African Americans and Caucasians at each cut point, using Caucasians with BLL < 5 μg/dL as the comparison group, allowing direct comparison of the risk estimates.

## Results

Table 1 shows the characteristics of the study population. Of the 31,526 children born between 1 January 1994 and 31 December 1997 and enrolled in the MCO, 4,634 had lead screening results in the laboratory database and were recorded as being African American or Caucasian. Children with lead screening results differed demographically from children without lead screens in terms of sex (fewer males in study sample; *p* = 0.02), race (more African Americans in study sample; *p* < 0.001), and median annual income (lower income for children in study sample; *p* < 0.001). The latter was observed regardless of race.

The percentages of children with BLL ≥ 5 μg/dL and ≥10 μg/dL were 39.0 and 8.6%, respectively. The overall mean BLL for the entire sample was 4.7 μg/dL (SD = 4.0, median = 4.0 μg/dL). African Americans had a higher mean BLL when compared with Caucasians, 5.5 μg/dL (SD = 4.3, median = 4.0 μg/dL) versus 3.2 μg/dL (SD = 2.5, median = 3.0 μg/dL), respectively, *p* < 0.01.

The period prevalence of asthma at baseline was 7.5% for definition 1 and 2.4% for definition 2 (Table 1). The period prevalence of definition 1 asthma among African Americans at baseline was 8.9% compared with 4.1% for Caucasians, *p* < 0.01 (Table 2). The period prevalence of definition 2 asthma among African Americans was 2.6% compared with 1.8% for Caucasians, *p* = 0.12.

The ID for the entire cohort was 2.4 and 0.9 per 100 person-years for definition 1 and definition 2 asthma, respectively. The ID of definition 1 asthma among African Americans was 3.0/100 person-years compared with 1.2/100 person-years for Caucasians, *p* < 0.01. The ID of definition 2 asthma among African Americans was 1.1/100 person-years versus 0.4/100 person-years for Caucasians, *p* = 0.01.

Table 2 shows a univariate analysis of the association of study variables to asthma prevalent and incident cases. African-American race, male sex, birth weight ≤2,500 g, and annual income ≤$10,027 (the median income per person for the study population) were significantly related to prevalent asthma (all *p*-values < 0.01). When compared with the reference group of BLL < 5 μg/dL, the OR (95% CI) for prevalent asthma was 1.2 (0.9–1.5), *p* = 0.14, for BLL ≥5 μg/dL, and 1.0 (0.7–1.6), *p* = 0.87, for BLL ≥10 μg/dL. African-American race, male sex, and birth weight ≤2,500 g were significantly associated with incident asthma (all *p*-values < 0.01). The HR for BLL ≥5 μg/dL and incident asthma was 1.2 (1.0–1.6), *p* = 0.09 and for BLL ≥10 μg/dL was 1.0 (0.6–1.6), *p* = 0.97.

Results of Cox proportional hazards analysis are shown in Table 3. All analyses were adjusted for annual income ≤$10,027, birth weight, and sex. Among Caucasians, the adjusted HR (adjHR) for definition 1 asthma was only slightly elevated for BLL ≥5 μg/dL and was not statistically significant (adjHR = 1.4; 95% CI, 0.7–2.9; *p* = 0.40). Again, there was no association between BLL ≥10 μg/dL and asthma. The risk estimate for definition 2 asthma was elevated for Caucasians with BLL ≥ 5 μg/dL but did not reach statistical significance (adjHR = 2.7; 95% CI, 0.9–8.1; *p* = 0.09). Among African Americans, BLL was not associated with developing definition 1 or 2 asthma.

We also conducted the Cox proportional hazards analysis for the association of BLL to incident asthma, using Caucasians with BLL < 5 μg/dL as reference (Table 4). Results were similar to that shown in Table 3, in that the adjHR for developing definition 1 asthma for Caucasians with BLL ≥5 μg/dL was elevated, but not significant, and BLL was not associated to incident asthma among Caucasians with BLL ≥10 μg/dL when compared with the reference group. AdjHRs (95% CIs) for African Americans with BLL < 5 μg/dL and BLL ≥5 μg/dL were 1.6 (1.4–2.0) and 1.4 (1.2–1.6), respectively, when compared with the reference group (both *p* < 0.01). At BLL ≥10 μg/dL, the adjHR (95% CI) for risk of asthma for African Americans was 2.1 (1.2–3.6), *p* = 0.01, for definition 1 and 3.0 (1.2–7.1), *p* = 0.01, for definition 2.

## Discussion

Lead poisoning and asthma jeopardize the health and quality of life of urban minority children in the United States (Bernard and McGeehin 2003; Lanphear et al. 2002). We sought to evaluate the contribution of BLL to the increased risk of asthma among African Americans. BLL was less a predictor of asthma than was race and did not affect the relationship of race to prevalent or incident asthma. Because lead poisoning and asthma share risk factors that are heavily influenced by SES, it is difficult to obtain an unbiased estimate of the true relationship (Bernard and McGeehin 2003; Lanphear et al. 1996; Needleman 1998). Previous studies have shown an association between BLL and serum IgE, and because serum IgE is observed in both atopic and nonatopic asthma, it was of interest to determine whether a relationship between BLL and development of asthma could be demonstrated using secondary data sources. To our knowledge, there are no studies that have looked at BLL and the incidence of asthma by race.

We observed an elevated risk of asthma among children exposed to lead, although these associations were not always statistically significant and were observed only for certain subgroups. Three interesting findings can be garnered from this study. First, a trend toward elevated risk estimates for asthma was observed for BLL at a cut point lower than what is currently considered toxic (Bernard and McGeehin 2003; Burns et al. 1999; Needleman and Landrigan 2004). Second, in addition to a trend toward increased risk at lead levels ≥5 μg/dL, the elevated risk was observed consistently only for Caucasians. Although the risk of developing asthma was significantly increased for African Americans when compared with Caucasians with BLL < 5 μg/dL, the risk was not dependent on BLL; that is, African Americans with BLL < 5 μg/dL were also at increased risk of asthma. Third, although our results are inconclusive regarding a dose–response relationship for BLL and asthma, among African Americans BLL ≥10 μg/dL held a higher risk of asthma than did BLL ≥5 μg/dL. Among Caucasians, the adjHR for BLL and incident asthma increased as the asthma definition became more stringent. However, because BLL is an inadequate dosimeter of lead exposure, a dose–response relationship between BLL and asthma may not be observed in our data, if such a relationship exists.

The trend toward an elevated risk of asthma observed among Caucasians with BLL ≥5 μg/dL could be a residual effect of factors unadjusted for in our analysis. If so, these risk estimates may indicate the presence of environmental exposures related to both BLL and asthma. Because the baseline BLL in this study could have been measured as late as 3 years of age, exposure to factors related to asthma may have already occurred. If this is true, BLL ≥5 μg/dL recorded during early infancy could be an indicator that risk factors for asthma are also present in the environment. There is growing evidence that exposures and events occurring during the first year of life are important determinants of the development of atopy and asthma (Holt 1998; Johnson et al. 1996 Johnson et al. 2002; Joseph et al. 2002; Ownby et al. 2002).

The racial differences observed are of interest. It was clear that African Americans were at a significantly higher risk of developing asthma when compared with Caucasians, regardless of BLL. The effect of BLL on the immune system of African-American children may be masked by more influential factors working to increase risk (Holt 1998; Joseph et al. 2000, 2002). Again, BLL may signal the presence of indoor environmental risk factors for asthma that play a greater role in development of the disease for African Americans. Racial differences in factors related to asthma, both environmental and otherwise, have been previously reported. Differential sensitization for indoor and outdoor allergens by race has been documented in at least two studies (Celedon et al. 2004; Joseph et al. 2000). Another possible explanation is the racial difference observed in IgE (Joseph et al. 2000; Oettgen and Geha 1999). In a previous study, we found that total IgE was higher for African Americans when compared with Caucasians among children with and without asthma, and that total IgE in African Americans was not related to bronchial hyperresponsiveness, despite the observed association in Caucasians (Joseph et al. 2000). Perhaps Caucasians are more sensitive to the effect of low levels of lead, whereas the BLLs studied were not high enough to induce an effect in African Americans.

Differences in lead sources may explain variations observed. A study conducted by Lanphear et al. (1998) reported differences in housing conditions and exposures to lead-contaminated house dust that contributed to observed racial differences in BLL. Although lead-contaminated soil was a risk factor for both racial groups, African-American children were more likely to be exposed to indoor environmental sources of lead (e.g., lead-contaminated house dust, painted surfaces, and floors in poor condition), whereas outdoor sources were more likely for Caucasian children (Lanphear et al. 1998).

Genetic variation may explain racial differences in susceptibility to lead poisoning. The C282Y mutation in the *HFE* gene causing hemochromatosis, and the gene coding for δ-aminolevulinic acid dehydratase, an enzyme of heme synthesis, are both associated with increased lead absorption. The vitamin D receptor gene can lead to increased production of calcium-binding proteins, also resulting in increased lead absorption. These genetic variations have not been shown to explain racial differences in lead toxicity (Lanphear et al. 1996; Onalaja and Claudio 2000; Wright et al. 2004).

The role of environmental lead in the development of atopic asthma is hypothesized to be mediated through IgE. The division of asthma into two clinical variants based on atopy continues to be controversial, but high total IgE is actually characteristic of both groups (Beeh et al. 2000; Romanet-Manent et al. 2002). It has been proposed that lead acts to increase production of IgE through direct or indirect stimulation of B-cells or through the binding and subsequent alteration of allergens that stimulate the allergenic immune response (Annesi-Maesano et al. 2003; Lutz et al. 1999). Several studies report an association between lead and IgE, but we found only one study exploring the relationship between lead and an asthma diagnosis: The study by Bener et. al. (2001), conducted in United Arab Emirates, found that industrial workers had significantly higher BLL (77.5 μg/dL, SD = 42.8) compared with non-industrial workers (19.8 μg/dL, SD = 12.3) and that the former also had a higher prevalence of asthma and respiratory symptoms.

Lead levels below those recognized as unsafe have been shown to inhibit production of interferon-γ , a TH_1_ immune response, and enhance TH_2_ responses [e.g., interleukin (IL)-4, IL-5, IL-10, IL-13, and IgE)] (Annesi-Maesano et al. 2003; Miller et al. 1998). Results of a laboratory study by Snyder et al. (2000) found evidence for maternal transfer of lead both transplacentally and lactationally in pregnant BALB/c mice and their offspring. The authors found that mouse neonates exposed to lead transplacentally and/or lactationally had significantly higher plasma IgE levels. Higher IgE levels among individuals exposed to lead have been corroborated in several human studies. Lutz et al. (1999) conducted a study of BLL and IgE in a predominantly Caucasian study population of 279 young children participating in the WIC (Women, Infants, Children) Nutritional Support Program and selected lead prevention programs active in Greene County, Missouri during the study period. In this study, BLL was significantly and positively associated with serum IgE levels. No relationship between cytokines measured in the blood and BLL was observed. Boscolo and colleagues (1999 (2000) examined the role of trace metals, including lead, in expression of lymphocyte subpopulations and cytokine serum levels in asymptomatic, atopic urban men and women. Atopy was defined as “evident clinical history of allergic symptoms.” In men, blood lead (mean BLL = 11 μg/dL) had an immunomodulatory effect on CD4^+^ and B-lymphocytes that appeared to enhance the production of TH_2_-like cytokines and IgE (Boscolo et al. 1999). Women 19–49 years of age had slightly higher BLL among those that were atopic (median BLL = 64 μg/dL for atopic vs. 55 μg/dL for nonatopic), but although serum IgE levels were higher in atopic women, TH_2_-like cytokines and blood lymphocyte subpopulations did not differ significantly by atopic status (Boscolo et al. 2000). The authors suggested that differences in lead metabolism or hormonal secretion by sex may explain the dissimilar results. Sun et al. (2003) conducted a study in a small number of preschool children (*n* = 73) in the People’s Republic of China. Overall, serum concentrations of IgE were higher in the high BLL group (≥10 μg/dL), but the association was of borderline significance (*p* = 0.069). When stratifying by sex, Sun et al. found that serum IgE levels were significantly higher only for females in the high BLL group (*p* = 0.027).

Limitations to this analysis include restricting this study to children enrolled in the MCO with results of lead screens in the laboratory database. This study population was more likely to be African American and had lower annual incomes per person than did those without a recorded BLL in the HFHS laboratory database. It is reasonable that African Americans and persons of low SES would be favored for lead screening in the MCO. According to the CDC and other sources, African-American and poor children in the United States are at a higher risk of lead poisoning when compared with Caucasian and with affluent children (Bernard and McGeehin 2003). The median BLL in this study (4.0 μg/dL) was higher than that reported for U.S. children 1–5 years of age (2.2 μg/dL) and for those with family incomes less than poverty level (2.8 μg/dL), according to national data for 1999–2000, indicating that children at risk are overrepresented in this cohort [U.S. Environmental Protection Agency (EPA) 2004].

Also excluded from this study would be children who received a lead screen using a finger or heel stick. In the laboratory database, lead levels are the result of venipuncture, which is considered more reliable than other methods (e.g., finger or heel stick). This was also a strength in that it permitted assessment of BLLs < 10 μg/dL.

Variables for analysis were limited to those collected in our hospital database. Consequently, there was no information on potential sources of lead. Parasitic infection is more prevalent among low SES groups, as is lead toxicity, potentially confounding a relationship between BLL and asthma, especially if the lead source is outdoors (Hagel et al. 2004). Moreover, we did not have information on other environmental exposures known to be associated with both asthma and BLL (e.g., environmental tobacco smoke, diesel exhaust) or other medical risk factors potentially associated with risk of asthma (e.g., family history of asthma or allergy, breast feeding, diet) (Johnson et al. 1996; Mannino et al. 2003). Using a definition of asthma based on encounters and prescription claims did not allow an investigation of differing asthma phenotypes, such as allergic asthma or transient wheeze (Martinez 2002; Romanet-Manent et al. 2002). Use of these databases, however, did allow a noninvasive exploration of the relationship of BLL and asthma in a population with both African-American and Caucasian representation. Using the MCO patient population may have reduced biases due to disparities in health care access, and having addresses allowed for geocoding that resulted in the ability to adjust for surrogate measures of SES (annual income per person).

We observed a trend toward an elevated risk of developing asthma in Caucasian children with evidence of BLL of ≥5 μg/dL before the age of 3 years. Assessment of the effect of BLL on IgE may provide a better understanding of the etiology and prevention of atopy and asthma. African Americans were at an increased risk of asthma when compared with Caucasians, but if there were any effects related to BLL, they were not observed. The racial differences observed in this study illustrate the need for further exploration of the role of race in the interrelationships between genetic susceptibility, socioenvironmental exposures, and risk of asthma.

## Figures and Tables

**Table 1:** Characteristics of the study population.

Characteristic	No.	Value
Age (years) at baseline BLL [mean ± SD (range)]	4,634	1.2 ± 0.5 (0.4–3.0)
Annual household income per person [US$ mean ± SD (range)]	4,450	10,579 ± 5,615 (1,819–47,077)
Sex		
Male	2,340	50.5
Female	2,294	49.5
Race		
African American	3,220	69.5
Caucasian	1,414	30.5
BLL (μg/dL)		
≥5	1,808	39.0
≥10	401	8.6
Period prevalence of asthma*^a^*		
Definition 1	346	7.5
Definition 2	109	2.4

Values are percentage except where otherwise noted.

a Asthma cases occurring from birth up to 12 months after the index BLL.

**Table 2:** Association of study variables to asthma period prevalence and incidence.*^a^*

	Prevalent asthma				Incident asthma*^b^*			
Variable	Asthma [*n* (%)]	No asthma [*n* (%)]	OR (95% CI)	*p*-Value	No. of cases	Person-years	HR (95% CI)	*p*-Value
African American	288 (8.9)	2,932 (91.1)	2.3 (1.7–3.1)	< 0.01	235	7,734	2.5 (1.8–3.4)	< 0.01
Caucasian	58 (4.1)	1,356 (95.9)			47	4,053		
Male	213 (9.1)	2,127 (90.9)	1.6 (1.3–2.0)	< 0.01	166	5,876	1.4 (1.1–1.8)	< 0.01
Female	133 (5.8)	2,161 (94.2)			116	5,912		
BW ≤2,500 g	63 (13.5)	405 (86.5)	2.1 (1.6–2.8)	< 0.01	38	1,054	1.5 (1.1–2.1)	< 0.02
BW > 2,500 g	277 (6.9)	3,754 (93.1)			240	10,427		
Income ≤$10,132*^c^*	206 (9.1)	2,054 (90.9)	1.6 (1.3–2.0)	< 0.01	138	5,218	1.1 (0.9–1.4)	0.35
Income > $10,132	128 (5.8)	2,062 (94.2)			139	6,205		
BLL < 5 μg/dL	198 (7.0)	2,628 (93.0)	1.0		166	7,639	1.0	
BLL ≥5 μg/dL	148 (8.2)	1,660 (91.8)	1.2 (0.9–1.5)	0.14	116	4,148	1.2 (1.0–1.6)	0.09
BLL ≥10 μg/dL	29 (7.2)	372 (92.8)	1.0 (0.7–1.6)	0.87	20	875	1.0 (0.6–1.6)	0.97

BW, birth weight.

a Definition 1 asthma; all persons with definition 2 asthma also fulfilled criteria for definition 1.

b Asthma cases per 100 person-years of enrollment ascertained during follow-up period (12 months postbaseline).

c Median income for the study sample.

**Table 3:** Results of Cox proportional hazards multivariable analysis of the association of BLL to incident asthma by race.*^a^*

Definition/race	BLL (μg/dL)	No.	With asthma [*n* (%)]	AdjHR (95% CI)	*p*-Value
Definition 1 asthma					
Caucasian	< 5	1,065	37 (3.5)	1.0	
	≥5	218	10 (4.6)	1.4 (0.7–2.9)	0.40
	≥10	27	1 (3.7)	1.1 (0.2–8.4)	0.91
African American	< 5	1,472	129 (8.8)	1.0	
	≥5	1,351	106 (7.9)	1.0 (0.8–1.3)	0.94
	≥10	322	19 (5.9)	0.9 (0.5–1.4)	0.58
Definition 2 asthma					
Caucasian	< 5	1,085	12 (1.1)	1.0	
	≥5	221	5 (2.2)	2.7 (0.9–8.1)	0.09
	≥10	28	0	—	
African American	< 5	1,580	51 (3.2)	1.0	
	≥5	1,444	43 (3.0)	1.1 (0.8–1.7)	0.53
	≥10	340	9 (2.7)	1.3 (0.6–2.6)	0.54

—, not calculated.

a Models adjusted for average annual income per person, birth weight, and sex. Separate models were created for ≥ 5 μg/dL and ≥10 μg/dL, both using < 5 μg/dL as the comparison group.

**Table 4:** Results of Cox proportional hazards multivariable analysis of the association of BLL to incident asthma using one race-exposure reference group.*^a^*

Definition/race	BLL (μg/dL)	No.	With asthma [*n* (%)]	AdjHR (95% CI)	*p*-Value
Definition 1 asthma					
Caucasian	< 5	1,065	37 (2.9)	1.0	
	≥5	218	10 (4.6)	1.4 (0.7–2.9)	0.33
	≥10	27	1 (3.7)	1.1 (0.1–7.7)	0.96
African American	< 5	1,472	129 (8.8)	1.6 (1.4–2.0)	< 0.01
	≥5	1,351	106 (7.9)	1.4 (1.2–1.6)	< 0.01
	≥10	322	19 (5.9)	2.1 (1.2–3.6)	0.01
Definition 2 asthma					
Caucasian	< 5	1,085	12 (1.1)	1.0	
	≥5	221	5 (2.2)	2.3 (0.8–6.7)	0.12
	≥10	28	0	—	
African American	< 5	1,580	51 (3.2)	1.8 (1.3–2.4)	< 0.01
	≥5	1,444	43 (3.0)	1.5 (1.2–1.8)	< 0.01
	≥10	340	9 (2.7)	3.0 (1.2–7.1)	0.01

—, not calculated.

a Models adjusted for average annual income per person, birth weight, and sex. Data represent five separate models, all using Caucasian with BLL < 5 μg/dL as the comparison group.
